# Long-Term Occupational Stress Is Associated with Regional Reductions in Brain Tissue Volumes

**DOI:** 10.1371/journal.pone.0064065

**Published:** 2013-06-11

**Authors:** Eva Blix, Aleksander Perski, Hans Berglund, Ivanka Savic

**Affiliations:** 1 Stockholm Brain Institute, Department of Women's and Children's Health, Division of Pediatric Neurology, Karolinska Institute, Stockholm, Sweden; 2 Stress Research Institute, Stockholm University, Stockholm, Sweden; University Children's Hospital, Germany

## Abstract

There are increasing reports of cognitive and psychological declines related to occupational stress in subjects without psychiatric premorbidity or major life trauma. The underlying neurobiology is unknown, and many question the notion that the described disabilities represent a medical condition. Using PET we recently found that persons suffering from chronic occupational stress had limbic reductions in the 5-HT1A receptor binding potential. Here we examine whether chronic work-related stress is also associated with changes in brain structure. We performed MRI-based voxel-based morphometry and structural volumetry in stressed subjects and unstressed controls focusing on gray (GM) and white matter (WM) volumes, and the volumes of hippocampus, caudate, and putamen – structures known to be susceptible to neurotoxic changes. Stressed subjects exhibited significant reductions in the GM volumes of the anterior cingulate cortex and the dorsolateral prefrontal cortex. Furthermore, their caudate and putamen volumes were reduced, and the volumes correlated inversely to the degree of perceived stress. Our results add to previous data on chronic psychosocial stress, and indicate a morphological involvement of the frontostriatal circuits. The present findings of morphological changes in these regions confirm our previous conclusion that symptoms from occupational stress merit careful investigations and targeted treatment.

## Introduction

Western societies are facing increasing reports of stress-related sickness among otherwise healthy and high-performing persons who report that they have not experienced any major negative life events or particular stress in early life [1], [2], [3], [4], [5]. These persons describe having stereotyped symptoms, including memory and concentration problems, sleeplessness, diffuse aches, profound fatigue, irritability, anxiety, and a feeling of being emotionally drained, which they often attribute to occupational stress. It has also been found that such stressed individuals can experience an acute phase with symptoms of hypertension, chest pain, dizziness and serious cognitive disabilities [6]. Even though many individuals recover from the acute symptoms, the cognitive and emotional dysfunction as well as the increased sensitivity to stress often last for months, or years, forcing the affected individuals to work part-time, change jobs or retire early. Many professionals are still not accepting the described disabilities as a medical condition, and when accepted, they tend to be misdiagnosed as depression. Only a minor portion of the affected individuals are helped by treatment with serotonin reuptake inhibitors or other antidepressants [7]. The major pathways of the physiological response to stress involve autonomic nervous system as well as hypothalamic–pituitary–adrenal (HPA) axis [8]. Although subjects suffering from symptoms attributed to chronic occupational stress are believed to have an altered reactivity of the HPA system, it is unknown whether this is a consistent finding [9], and both normal [10], [11], reduced [12], [13], [14], [15], and elevated [16], [17], [18] cortisol levels after awakening have been reported. Furthermore, in contrast to patients with major depression [19], subjects with chronic occupational stress symptoms show reduced cortisol and ACTH responses to the Corticotropin Releasing Hormone after dexametasone pretreatment [5], [20]. Emotional reactions to chronic stress and major depression, thus, seem to represent at least partly separate constructs, even if some symptoms (such as anxiety and attention and memory deficits) may overlap.

In a recent PET study, we found that patients suffering from chronic work-related psychosocial stress had significant reductions in the 5-HT_1A_ receptor binding in three limbic structures: the hippocampus, the anterior cingulate cortex (ACC), and the anterior insular cortex [21]. A functional disconnection was also found between the amygdala, the ACC, and the medial prefrontal cortex (mPFC), despite the absence of psychiatric co-morbidity and major negative life events [21]. The locations of changes, in several aspects, corresponded to the locations of structural changes detected through MRI in persons suffering from other stress-related conditions, such as stress in early life, repeated stressful negative life events, and post-traumatic stress disorder (PTSD), [22], [23], [24], [25], [26], [27], [28], [29].

This similarity raises the question of whether certain changes in structural volumes also occur among persons with symptoms related to chronic work-related stress. This is of interest for a couple reasons. Firstly, since the pathophysiology of symptoms attributed to occupational stress is highly debated, there is a need for clarification on whether this condition is associated with any cerebral changes. Secondly, if structural changes similar to those described in other stress-related conditions are also linked to occupational stress, one may hypothesize that chronic psychosocial stress affects our brains in a rather stereotyped manner, regardless of the underlying cause, and that cerebral changes are not limited to exposure to extreme and life threatening situations, but can also be related to accumulated everyday stress.

We therefore carried out comparative MRI studies of cerebral gray matter (GM) and white matter (WM) volumes between patients with chronic occupation-related stress and healthy controls. The study also included an analysis of the structural volumes of the hippocampus, caudate, and putamen. The two latter regions, to the best of our knowledge, have not been previously investigated in this population. They were, however, of interest because they have been shown to process stress stimuli [30], [31], [32], [33] Furthermore, magnetic resonance imaging (MRI) of a large sample of adults with no history of psychopathology revealed that people who have experienced significant early life stress have volumetric reductions in the caudate nucleus in addition to the anterior cingulate cortex [24]. Also, a recent study of effects of life traumas during youth, reveal reductions in the caudate and putamen volumes [34], further emphasizing that volume changes in these structures may be related to stress. On the basis of these previous data, and assuming that a repetitive stress-induced activation could lead to neuronal and dendritic damage in the structures involved, it was hypothesized that the caudate and putamen volumes may be smaller in persons suffering from prolonged occupational stress than in controls. Given previous findings on the effects of stress on the brain [5], [6], [21], [35], a further hypothesis was that our stressed subjects would have reductions in the GM and WM volumes in the ACC and mPFC, and in the grey matter volumes of the hippocampus, amygdala and the insular cortex. These hypotheses were tested in a magnetic resonance (MR) study, which combined two different analyses – voxel based morphometry (VBM) and structural volumetry. VBM was carried out in two ways: 1) exploratively, with the entire brain as search space, to investigate whether there were any regional GM and WM changes in the brain on pixel by pixel basis. 2) It was also used with the assumption that GM and WM would be changed in regions known to be consistently involved in the regulation of stress (such as the hippocampus, mesial prefrontal cortex, insular cortex and the amygdala), and therefore, restricting the search space to a mask covering these areas (see methods). Structural volumetry was carried out in addition to the VBM analyses because some subcortical structures, basal ganglia in particular, have a poor white and gray matter demarcation, and the volumes of these structures are better defined with manual delineation.

## Methods

### Subjects

Thirty right-handed [36], non-smoking patients (23 women and 7 men, age 41.3±6.6, range 36–55 years, education 13.5±2 years), who had been diagnosed as having had a ‘reaction to severe stress and an adjustment disorder’ according to the International Classification of Diseases (ICD-10, F43), were recruited from the Stress Research Institute at Stockholm University. In order to compose a study group with a homogenous etiology and to reduce variability, the selection was limited to subjects who attributed their illness to prolonged work-related stress, after working 60 to 70 hours per week continuously over several years prior to the onset of symptoms. Inclusion criteria consisted of a characteristic symptom course of sleeplessness, diffuse aches, palpitations and fatigue, a subsequent onset of irritability, anxiety, memory and concentration problems, feeling of depersonalization, and reduced work capacity (confirmed by the employers) [5], [6]. All of the subjects attributed their symptoms to chronic stress and had no other known etiology for their distress.

Subjects were also required to have had a symptom duration of at least one year, to have been on sick leave (≥50%) for stress-related symptoms for a minimum of 6 months before entering the study, and to have an average stress-burnout score of *≥3.0* on the Maslach Stress-Burnout Inventory – General Survey (MBI-GS), [37]. This 7-point rating scale, ranging from 0 (never) to 6 (daily), consists of three subscales: exhaustion (five items), cynicism (five items), and lack of professional efficacy (six items). When rating perceived stress, subjects were asked to take into consideration the last six months, and not only the actual time-point. The average scores for Scandinavian populations are around 2 for MBI-GS, [1], [38].

Subjects were excluded if they had previous history of psychosis, personality disorder, major or bipolar depression, alcohol or substance abuse, chronic fatigue, chronic pain, fibromyalgia, or neurological or endocrine disease. Subjects, who had experienced prominent stress factors in their private life or a major traumatic event at any time in their life, including sexual abuse, were also excluded. No daily medication was allowed during the two months prior to the study, except contraceptives. Review of past history of pharmacological treatment revealed no drugs known to affect brain structure (for example, psychopharmaca).

Sixty-eight healthy, right-handed, non-smoking volunteers (53 females and 15 males, age 37.5±7.2 years, range 27–51 years, education 13.0±2.35 years) with no history of chronic stress or heredity for neuropsychiatric disorders were used as the control group. The patient and control groups therefore had similar gender distributions, and both groups had a female dominance to accommodate with the female-dominated epidemiology of the condition studied [1]. The study was approved by the Ethics Committee at the Karolinska Institute, and written informed consent was received from each participant.

Before the interview, participants completed questionnaires in order to evaluate their stress symptoms and assess their previous life events. In addition, the occurrence of major life events among the subjects was assessed through a clinical psychiatric interview based on the non-work-related items of the Holmes and Rahe Scale [39]. The participants were asked to answer yes or no to whether they had experienced any non-work-related stressful life events (e.g., death of a relative or spouse, recent divorce, forced family relocation). Subjects were excluded if they answered positively to having experienced such an event in their lives. Patients also received a medical screening, (physical examination, test of thyroid and liver function). The possible presence of psychiatric disorders or personality disturbances were assessed according to the Diagnostic and Statistical Manual of the American Psychiatric Association, 4th Edition (DSM–IV), including the Structured Questionnaire for DSM-IV® Axis I and II (Structured Clinical Interview for DSM-IV® (SCID-I, and II) (American Psychiatric Publishing Inc, Arlington, 1997), along with a test for depression using the Montgomery-Asberg Depression scale [40].

### Magnetic Resonance Imaging

#### Data acquisition

All magnetic resonance imaging data was acquired on a whole-body 1.5-Tesla MRI medical scanner (General Electric, Milwaukee, Wisconsin) equipped with an 8-channel phased array coil. The MRI protocol included the following scans: 1) 3D-weighted T1 SPGR images with 1 mm isotropic voxel size according to a previously described protocol [41]; and 2) 2D T2-weighted fast spin echo (FSE) images in the axial plane (effective TE = 56 ms, TR = 2500 ms, FOV = 24 cm, 23 slices of 3 mm thickness). The 2D images were not used in the present analysis.

#### Voxel-Based Morphometry (VBM)

We used a version of the VBM in the SPM5 package (www.fil.ion.ucl.ac.uk/spm) with the Gaser toolbox (http://dbm.neuro.uni-jena.de/vbm/) and Matlab 7.3 (Math Works, Natick, MA). The VBM pre-processing included five steps:

1. Check for scanner artifacts and gross anatomical abnormalities for each subject.
2. Set of image origin at the Anterior Commissure AC.
3. Using the Hidden Markov Random Field (HMRF) option in the segmentation of the VBM5 toolbox to minimize the noise level of the segmentation.
4. Using the Diffeomorphic Anatomical Registration Through Lie Algebra toolbox (DARTEL, Wellcome Department of Imaging Neuroscience, University College London, UK; http://www.fil.ion.ucl.ac.uk/spm) for a high-dimensional normalization protocol. We followed the standard version of John Ashburner's chapter including the MNI space transformation [42].
5. To restore the original volume information within each voxel, voxel values in the segmented images were modulated (multiplied) by the Jacobian determinants derived from the spatial normalization step. The analyses of modulated data allowed direct comparisons of regional differences in the amount of each tissue type.

After pre-processing, visually checking for homogeneity across the sample, smoothed (8 mm), modulated, and normalized images were obtained and used for the statistical analyses. These modulated volume images of the respective tissue types are hereafter referred to as GM, WM, and cerebrospinal fluid (CSF).

#### Structural volumetry

In regions with poor white and gray matter demarcation, as in basal ganglia, volumetry is regarded as more reliable than VBM analysis [43], and manual volumetry was, therefore, used in addition to VBM. Homologous VOIs were delineated manually for the hippocampus, caudate, and putamen in all the subjects.

All the volumes were delineated on original, un-reformatted T1 images using MRIcro software (www.sph.sc.edu/comd/rorden/mricro.html) by two investigators who were uninformed about the identity of the subjects. Values presented in *Results* and Table 1b were generated by investigator 1, who analyzed all the data (investigator 2 analyzed structural volumes from 15 randomized subjects in each study group). Both raters analyzed 10 images twice to establish the intra-rater reliability. VOIs were outlined according to previously described protocols [43], [44], [45]. In summary.

**Table 1:** Demographics.

	Patients (n = 30)	Controls (n = 68)	P and F values
Age (years)	41.3±6.6	37.5±7.2	p = 0.378 F = 0.783
Education (years)	13.5±1.8	13.0±2.4	p = 0.423 F = 0.648
MBI- GS (score)	4.2±1.1	2.2±0.5	p<0.0001 F = 100.6
MADRAS (score)	10.6±6.3	7.3±3.9	p = 0.12 F = 2.58

Age and education are expressed in years; MBI-GS is a questionnaire to score work-related stress symptoms. MADRAS  =  Montgomery Asberg Depression Scale.

The hippocampus*:* the hippocampus was traced according to the protocol of Watson et al. [46]. The posterior boundary of the hippocampus was defined as the first image in which the crus of the fornix became visible. The superior boundary consisted of the alveus; the lateral boundary was the inferior corn of the lateral ventricle; the ambient cistern was the medial boundary, whereas the WM of the parahippocampal gyrus represented the inferior boundary. The hippocampus VOI included the tail of the hippocampus.

The caudate nucleus: the caudate nuclei were traced separately, in accordance with Raz et al. [47]. The lateral ventricle was used as the medial boundary; the subcallosal fasciculus served as the anterior boundary; the stria terminalis was the posterior boundary (identified by a change in signal intensity relative to the caudate nucleus); and the anterior limb of the internal capsule served as the lateral boundary. The region of interest included the head, the body of the caudate, and the tail (excluding the portion that turned anterior). The nucleus accumbens was excluded as well.

The putamen: the delineation of the putamen started in the first slice where it became visible laterally to the caudate. The superior boundary was defined by the corona radiata, the internal capsule was the medial border, and the lateral border was the external capsule. The posterior limit was the point at which the putamen was no longer visible in the corona radiata. The lower limit was just above the amygdala, excluding nucleus accumbens, when the operator could see the most inferior aspect of the third ventricle and the chiasmatic cistern was no longer visible.

The hippocampus was delineated on coronal images, and the other structures on horizontal sections. All the volumes were then viewed and corrected on coronal, horizontal, as well as sagital images.

### Statistical analysis

Group differences in age and education, as well as differences in the total intracranial volume (TIV), calculated as the total volume of GM + WM + CSF, and the total tissue volume (TV), calculated as the total volume of GM + WM, were tested with unpaired Students t-test (p<0.05). Group comparisons of the total GM and WM volumes were tested using analysis of covariance (ANCOVA, p<0.05), taking into consideration individual differences in TIV, which was used as the covariate in addition to age and sex (the latter was not strictly necessary as the groups were matched for sex). Group comparisons of relative structural volumes (VOI/TIV) were carried out with unpaired Students t-tests using the mean individual relative values of the two homologous VOIs for each type of structure as input values (p<0.016), after Bonferroni correction for the three separate regions). The aforementioned analyses were carried out with PASW Statistics 18 (SPSS Inc., Chicago, IL).

**Figure 1:**
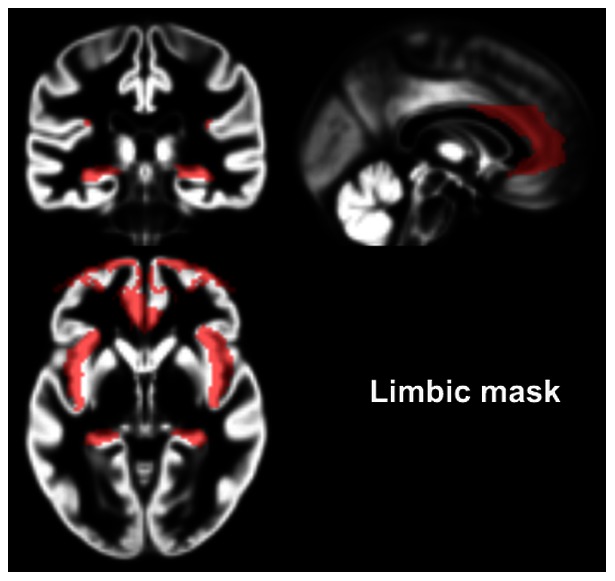
Illustration of the limbic mask, constructed from the WFU-pick atlas (please see methods), superimposed on the mean grey matter images from the entire study population.

Group differences in GM and WM volumes within the regions that were expected to show changes in stressed subjects (the ACC and mPFC, the hippocampus, amygdala and the insular cortex) were tested with VBM by restricting the search space to a mask encompassing the amygdalae (both sides), hippocampi, the ACC, the medial and superior frontal gyrus, and the insular cortex (both sides). This mask was derived using the WFU Pick Atlas (maldjian@wfubmc.edu), by adding the respective regional areas as defined by the atlas into a confluent, large mask (Fig. 1). In addition, we employed explorative analysis using also the entire brain as the search space to investigate possible differences between the two subject groups in other regions. Significant clusters were defined with flexible factorial design in SPM5 (voxel threshold p<0.001, with FDR correction at p<0.05) using age, sex, and the total brain volume (TIV) as covariates of no interest. We also implemented non-stationary cluster extent correction in the Gaser toolbox to correct for the non-uniform smoothness in the VBM images.

The co-ordinates are reported in Montreal Neurological Institute (MNI) space.

We hypothesized that changes in regional GM and WM, and in the structural volumes would be related to the degree of perceived stress. To test this, correlation analyses were carried out between MBI-GS scores and the individual GM or WM volumes extracted from the clusters showing significant group differences in GM and WM volumes as well as the relative volumes (the VOI/TIV ratios) of structures showing a significant difference between patients and controls. For the correlation analyses between VOI/TIV and MBI-GS, we used the mean of the two homologous regions (provided that a possible difference from controls was bilateral) in order to reduce the number of comparisons. The significance value for these linear regressions was set at p<0.05, because each of the regions included in this calculation was assumed to be affected by stress.

Inter- and intra-rater reliability was tested with linear correlation analysis, Pearson's coefficient, p<0.05.

## Results

### Clinical data

The groups did not differ in age or education. (Table 1).

No significant difference was detected between the two subject groups in respect to depression, as assessed by MADRAS scores (p = 0.12), although the mean value was higher among patients (Table 1). However, the MBI-GS scores, indicating perceived work-related stress levels, were significantly higher among the patients (4.2±1.1 vs. 2.2±0.5; p<0.0001; F = 100.6, df = 1); among controls, the scores were below 3.0 and in the range reported in other studies [1], [38], (Table 1).

No gross anatomical abnormalities were found, as judged by an experienced neuroradiologist, and none of the subjects had to be excluded due to either movement artifacts during scanning or segmentation errors.

We did not detect any group difference regarding total GM volume, WM volume, total TV, or the TIV (Table 2).

**Table 2:** Structural volumes.

Volumes (cm^3^)	Patients	Controls	P and F values
L caudate volume	3.8±0.5	4.6±0.6	p<0.0001 F = 26.8
R caudate volume	3.8±0.5	4.5±0.6	p<0.001 F = 11.7
L putamen volume	3.8±0.5	4.6±0.7	p<0.0001 F = 23.4
R putamen volume	3.8±0.4	4.7±0.6	p<0.0001 F = 25.8
L hippocampus volume	3.0±0.4	3.0±0.4	p = 0.312 F = 1.054
R hippocampus volume	3.0±0.4	3,0±0.5	p = 0.493 F = 0.483
GM volume	690.3±57.9	699.2±66.4	p = 0.208 F = 1.608
WM volume	458.9±50.4	457.5±48.4	p = 0.88 F = 1.148
CSF volume	338.4±100.9	457.5±48.4	p = 0.86 F = 0.033
TIV volume	1487.6±163.3	1490.5±151.3	p = 0.57 F = 0.331

TIV  =  total intracranial volume.

P-values for structural volumes were based on calculations of ratios between the respective structural volume and the TIV.

### Structural volumetry

Patients showed a significantly lower structural volume relative to the TIV (VOI/TIV) bilaterally in the caudate and putamen (Table 2). The results remained when adding age and MADRAS as covariate (p<0.001 for both structures, post hoc analysis with ANCOVA). No significant group difference was detected in the volume of the hippocampus. The inter-rater correlation was 0.8 for the measurements of the hippocampus, and 0.9 for the caudate and putamen; the corresponding intra-rater values were 0.8, 1.0, respectively.

### Voxel-based morphometry

**Figure 2:**
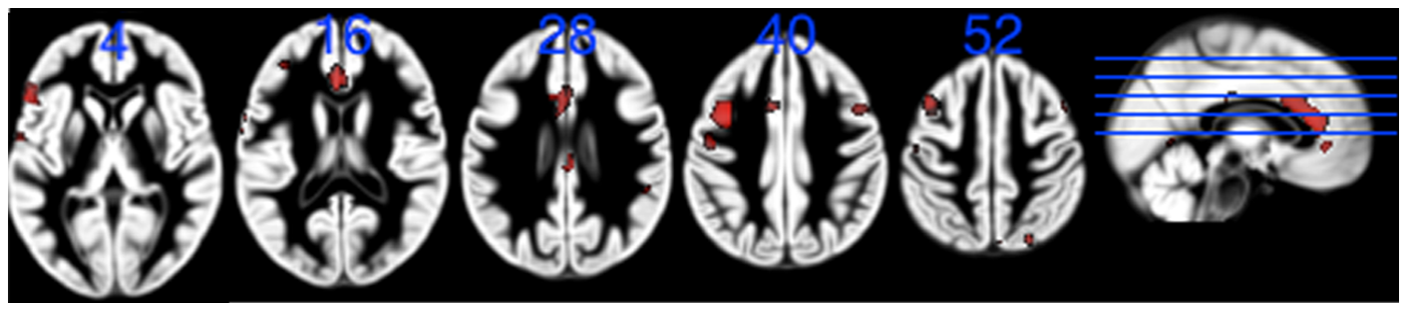
Reductions in GM volumes in stressed subjects. Significant reductions in the GM (red) in stressed subjects compared with controls. Clusters calculated using peak threshold at p = 0.001, FDR corrected at p<0.05. All the clusters are superimposed on the GM template from the entire study group. The numbers indicate z-levels in MNI co-ordinates. R = right side.

Significant reductions in the GM volumes were detected in three clusters among the patients. They were located in the ACC (a region covered by the a priori hypothesis), and, in addition, in the left and right middle frontal gyrus (regions constituting portions of the dorsolateral prefrontal cortex – dlPFC), (Table 3, Fig. 2). Contrary to the hypothesis, we found no changes in the hippocampus, the amygdala, or the anterior insular cortex, (Table 3). No regional increases in GM were found in among patients. No group differences were detected in regional WM.

**Table 3:** Significant group difference in GM volume.

	Controls > Patients			Patients > Controls
	Z-level	Size (cm^3^)	Coordinates	
Left middle frontal gyrus	4.3	1.9	25–11 59	
Anterior cingulate cortex (BA 32) #	3.4	0.5	−39 6 46	**NONE**
Right middle frontal gyrus	5.0	2.1	−2 31 13	

# Cluster showing a significant group difference when using a limbic mask comprising the anterior cingulate cortex, the mPFC, the insular cortex, the hippocampus and the amygdala, using peak threshold at p = 0.001, FDR corrected at p<0.05. The other clusters were calculated with same level of significance, but using the entire brain as search space (no a priori hypothesis).

No group differences were observed in white matter volumes and no significant clusters were detected when reversing the contrast (using the contrast: stressed patients – controls).

Post hoc group comparison when adding MADRAS as covariate of no interest did not alter the results.

### Correlation analyses

**Figure 3:**
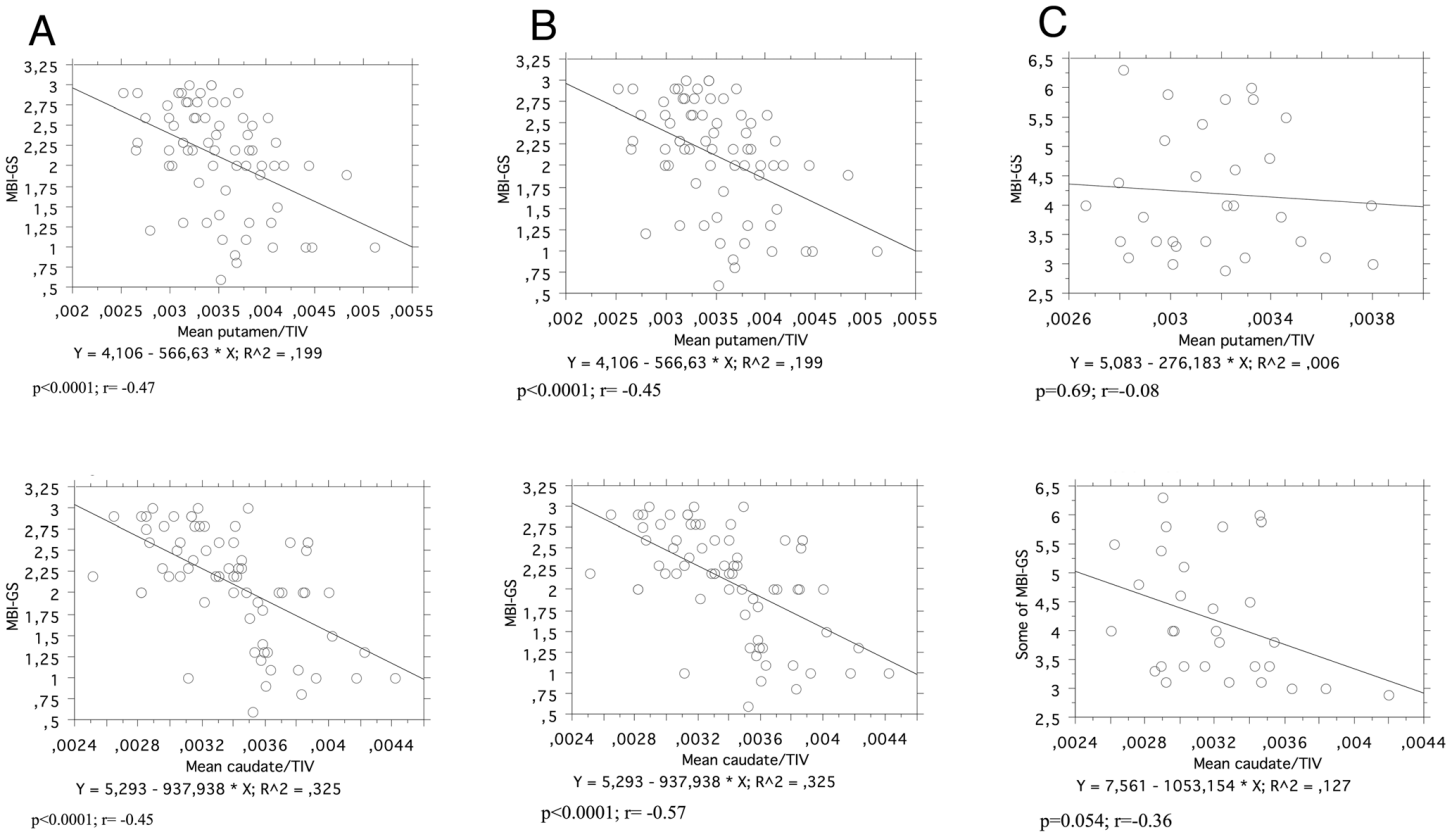
Correlation analysis between stress scores (MBI-GS) and the relative putamen volume (mean of the right and left putamen/TIV) – upper panel, and the relative volume of the caudate (mean of the right and left caudate/TIV) – lower panel. A = All subjects; B =  Control subjets only; C = Stressed subjects only.

No correlations were detected between stress scores (MBI-GS) and the individual GM volumes extracted from the clusters showing significant differences between controls and patients (the ACC, the left and right middle frontal gyrus). There was, however, a statistically significant, negative correlation between the relative structural volume of the caudate and putamen (mean of the right and left values for the respective structure) and the MBI-GS scores (r = −0.47 and p = 0.0001, r = −0.45 p = 0.0001, respectively), Fig. 3A. When estimating the regression lines separately for stressed subjects and controls, significant inverse correlations remained amongst the controls for both structures (r = −0.57, and r = −0.45; p<0.0001 for both), Fig. 3B, whereas amongst the stressed group there was only a tendency for an inverse correlation in the caudate (r = −0.36, p = 0.054), and no correlation with the relative volume of the putamen (Fig. 3C).

## Discussion

The present study tests the hypothesis that perceived prolonged occupational stress is associated with regional morphological changes in the brain. Such changes have been found in patients with PTSD (thus, in relation to life threatening trauma), in persons having experienced early life traumas, and in those reporting negative major life events [23], [27]. However, to the best of our knowledge, the possibility of such changes has not been previously discussed in relation to subjects undergoing occupational stress who have not faced extreme life conditions or psychosocial trauma and who have no history of psychiatric disorders.

The principal findings were the reductions in the GM volumes of the ACC and the dlPFC, and reductions in the volumes of the caudate and putamen. The basal ganglia volumes were also inversely correlated with the degree of perceived stress. All the structures in which we detected changes have been described to be sensitive to stress stimuli [21], [23], [48], [49] and are known to be involved in stress physiology [31], [33], [50], [51]. Decreased GM volume and density in the prefrontal cortex and the ACC in particular has been detected in several studies of persons suffering from PTSD [26], [27], [52], [53], [54]. Of particular interest is the data of Kasai et al., showing atrophy of the ACC, hippocampus and insular cortex in combat veterans with PTSD but not in their identical twins, suggesting that the detected abnormality was acquired [26]. Furthermore, measurements of cortical thickness in a separate group of patients with PTSD have shown dynamic, stress-related changes in the dlPFC which were characterized by an increase in cortical thickness that gradually normalized over time during recovery [55].

Data from studies on basal ganglia in corresponding populations are less abundant. There are reports about an affection of the caudate and putamen in relation to early life traumas [56] and PTSD, [57], but in general, these structures have received relatively minor interest in the context of stress. The present finding of reduced structural volumes in the caudate and putamen, therefore, deserves particular consideration. This finding is in accordance with the reported activations of the basal ganglia occurring during acute stress [31], [58], [59]. It is also compatible with the notion that both the putamen and caudate receive powerful glutamatergic input from the prefrontal cortex [60] and are susceptible to excitotoxicity [61], [62]. The present findings are also congruent with the well-known phenomenon of stress-related freezing [63] and raises the question of whether and how motor performance may be affected in persons suffering from chronic psychosocial stress.

The regions showing changes in our stressed patients are part of a network believed to mediate the integration of cognitive, affective, and autonomic responses [64].

It is tenable that an affection of these structures could lead to poor attention, and working memory deficits, symptoms which individuals with chronic occupational stress have described experiencing [5], [6], [65]. The preset data is in line with results from a fMRI study by Sandström et al., showing a decreased activation of the dlPFC among 10 patients suffering from occupational stress [6], and with a fMRI study by Qin et al., which detected a reduced activation of the dlPFC in healthy subjects who performed a working memory task under acute stress [51].

Contrary to our hypothesis, but in accordance with the presently available reports from MRI studies of subjects with occupational stress [5], [21], no changes were detected in the hippocampus or amygdala. Among studies of other chronic stress conditions, relatively few have examined amygdala volumes, and the results are variable. Among persons with PTSD, smaller volumes have been reported in some studies [66], [67], while others have detected normal values [68], [69], [70]. Similar inconsistency appears with regard to the hippocampus [5], [69], [71].

One possible explanation for the mixed findings could be that the small size of these structures, the amygdala in particular, may lead to greater variability in volume measures, which hampers the probability of detecting significant group differences, especially when the investigated study groups are undersized. Given the size of the present population and the use of 1.5 Tesla scanner, we did not try to specifically assess the structural volume of the amygdala in the present study. Another potential explanation may be associated with time of the stress exposure in relation to age as such exposure may have effects on these structures under sensitive developmental periods [72], [73].

The fact that the presently detected volume reductions were confined to regions believed to be involved in the processing of stress stimuli [23] makes it highly unlikely that they were random findings. The threshold used in the SPM analysis was in accordance with other VBM studies of psychosocial stress in humans [27], [74], [75] and the detected differences in the structural volumes of the caudate and putamen were highly significant.

At present, we can only speculate about the underpinnings of the observed changes. Because the study was cross-sectional, it is difficult to state whether the detected reductions represent the neurotoxic effect of stress, effect of other factors, such as nitric oxide, or are associated with a pre-existing condition that could have rendered the brain more vulnerable to the development of pathological stress responses. Due to our strict selection criteria, it is, however, possible to exclude potential confounding factors such as major life traumas, psychiatric premorbidity, depression, chronic pain, and pharmacological treatment. Considering the congruence with data from animal experiments as well as with the longitudinal data from patients with PTSD, we find it probable that the present findings reflect effects of chronic psychosocial stress, in this case, occupational stress. The observed inverse correlation between the stress scores and the relative volumes of the caudate and putamen (Fig. 3) could be taken as an argument for this view. It should be notified, however, that this correlation is dominated by the data from controls (r = −0.57, p = 0,0001, and r = −0.45, p = 0.0001, whereas the corresponding regression line in stressed subjects only showed a non-significant trend for the caudate (r = −0.36 p = 0.054), and no trend for the putamen (r = −0.08; p = 0.69). The reason for that is not evident, one possibility could be that the control group was much larger, and that the spread of volumes was larger among the controls. Worth mentioning is that in our recent follow up investigation of two new cohorts of controls and subjects with occupational stress, shows very similar results (Osika&Savic, manuscript in preparation).

The molecular underpinnings of the morphological changes after stress are just beginning to be studied in detail, please see Leuner and Shors 2012, and Conrad 2008, for a comprehensive review [76], [77]. The major mediators of stress-related neuronal modulation involving dendritic retraction and in some cases neurotoxicity are glucocorticoids and glutamate [23]. They have own neuronal effects, and are also reported to interact [78], [79]. Circulating glucocorticoids interact with various neurotransmitters, [80] and chronic stress in tree shrews is found to reduce the number of dopamine transporter (DAT) binding sites (B_max_) in the caudate nucleus and the putamen [81]. Adrenal ectomy is reported to increase the proliferation of hippocampal neurons whereas excess in glucocorticoid is reported to decrease it sharply, as do psychosocial stressors [82]. Experiments with betamethasone infusion in fetal sheep show glucocorticoid-related loss of synaptic density in the frontal neocortex, caudate, putamen, and hippocampus [83], [84]. While these alterations were initially viewed as a neurodegenerative event, it is worth mentioning that more recent studies suggest that stress induced dendritic alterations are reversible if animals are given time to recover from chronic stress [77].

The second stress-associated factor, which could have contributed to the observed changes, is glutamate. Data from animal experiments show that stress causes an enhanced release of glutamate, and that a stress-related elevation of extracellular glutamate levels induces retraction in the spines in stress-targeted regions, such as the mPFC, ACC, and the basal ganglia [79], [85], [86], [87], [88]. Medial prefrontal cortex is reported to be sensitive to repetitive stress [89]. See also the review by Leuner and Shors [77]. Via glutamatergic excitation the mPFC and ACC send inhibitory GABA-ergic impulses to the amygdala, which is the primary cerebral relay for the processing of psychosocial stress stimuli [90]. One possible scenario is that the absence of prefrontal inhibition of the amygdala, due to the stress-mediated neurotoxic damage of the mPFC (due to high glutamate, cortisol or the combination of both [79], [91], may cause amygdala hyperactivity [92], providing a context for a vicious circle with increased excitation and excitotoxic changes along the networks linked to the amygdala and mPFC. These networks primarily include the dlPFC, the basal ganglia, and the limbic brain (the hippocampus and insular cortex). Whereas the ACC, the dlPFC, and the basal ganglia showed distinct changes, neither the hippocampus nor the insular cortex was seemingly affected according to our results, even though these regions were included in the specific search space defined by our limbic mask. This was unexpected, especially when considering that both structures had significant reductions in 5-HT_1A_ receptor binding potential in our previous study of a similar population [21]. The underlying reasons are not clear, and a larger population of subjects needs to be investigated before this lack of significance can be taken as an argument for specificity of the previously detected 5-HT_1A_ receptor changes.

### Methodological limitations and strengths

Several methodological limitations deserve comment. Firstly, the sample was rather small even though the sensitivity was enhanced by the use of a homogenous study group. Secondly, causality cannot be determined in the present study, and longitudinal studies would be needed to provide a better understanding of the temporal relationships between morphological brain alterations and periods of psychosocial stress. The issue of comorbidity with depression is always a problem in a study group like our – as some symptoms are overlapping and the condition of profound fatigue and inability to lead a normal life is perceived as extremely taxing by some subjects. The stressed subjects were not deemed as depressed by the experienced psychiatrist. We also re-run the comparisons between groups using MADRAs scores as covariate in addition to age and gender when comparing the mean relative caudate and putamen values between the groups. The results remained (p = 0.0061 for caudate and p = 0.0002 for putamen). Also in the VBM analysis, when controlling for depression by entering depression scores as covariate the results remained unchanged. Finally, neuropsychological data was not collected systematically, as we whished to investigate weather there are any anatomical changes in the brain that could be related to occupational stress, before designing a study to test possible behavioural correlates. Such study is on going.

The advantage of the present study is that it combines VBM analysis and investigations of structural volumes. In regions with poor white and gray matter demarcation, as in the basal ganglia, volumetry is regarded as more reliable than VBM [43], and the two methods should, therefore, be used in tandem. Consequently, it is not surprising that structural volumetry showed reductions in the basal ganglia while the corresponding GM and VM volumes did not differ from controls.

## Conclusions

By investigating the GM and WM volumes and structural volumes of subjects who are experiencing chronic psychosocial stress, but do not have a history of a particular psychosocial trauma, the present study expands upon the data from animal experiments and reports from PTSD patients, and focuses on the involvement of the frontostriatal circuits in chronic stress. The finding of GM atrophy in regions known to be associated with chronic psychosocial stress confirms our previous conclusion that subjects reporting stereotyped symptoms from occupational stress have a medical condition requiring careful investigations and a targeted treatment. Larger series and consecutive investigations before and after treatments, as well as parallel mapping of cognitive and motor functions are highly encouraged in the future, and might have important implications for the understanding of this increasingly common condition.
